# Widespread nocturnality of living birds stemming from their common ancestor

**DOI:** 10.1186/s12862-019-1508-y

**Published:** 2019-10-16

**Authors:** Yonghua Wu

**Affiliations:** 1 School of Life Sciences, Northeast Normal University, 5268 Renmin Street, Changchun, 130024 China; 2 Jilin Provincial Key Laboratory of Animal Resource Conservation and Utilization, Northeast Normal University, 2555 Jingyue Street, Changchun, 130117 China

**Keywords:** Nocturnality, Phototransduction genes, Positive selection, Diel activity pattern

## Abstract

**Background:**

Many living birds exhibit some nocturnal activity, but the genetic basis and evolutionary origins of their nocturnality remain unknown.

**Results:**

Here, we used a molecular phyloecological approach to analyze the adaptive evolution of 33 phototransduction genes in diverse bird lineages. Our results suggest that functional enhancement of two night-vision genes, namely, *GRK1* and *SLC24A1*, underlies the nocturnal adaption of living birds. Further analyses showed that the diel activity patterns of birds have remained relatively unchanged since their common ancestor, suggesting that the widespread nocturnal activity of many living birds may largely stem from their common ancestor rather than independent evolution. Despite this evolutionary conservation of diel activity patterns in birds, photoresponse recovery genes were found to be frequently subjected to positive selection in diverse bird lineages, suggesting that birds generally have evolved an increased capacity for motion detection. Moreover, we detected positive selection on both dim-light vision genes and bright-light vision genes in the class Aves, suggesting divergent evolution of the vision of birds from that of reptiles and that different bird lineages have evolved certain visual adaptions to their specific light conditions.

**Conclusions:**

This study suggests that the widespread nocturnality of extant birds has a deep evolutionary origin tracing back to their common ancestor.

## Background

Living birds are generally considered diurnal, with the exception of few truly nocturnal species such as owls, nightjars and kiwis, but many diurnal birds exhibit partial or occasional nocturnal activity related to migration, dispersal, foraging, homing and singing [1, 2]. The presence of at least some form of nocturnal activity across almost all avian lineages has long been recognized and is well documented [1, 3, 4]. This nocturnal activity may have been facilitated by genetic adaptations [1, 2, 5]. Indeed, a night-vision-specific brain area has been identified in nocturnally migratory songbirds [6]. While recent studies show that nocturnal taxa frequently exhibit functional enhancement of dim-light vision genes involved in the rod phototransduction pathway [7–11], it remains unknown whether this is characteristic of the evolutionary history of living birds in light of their widespread nocturnality.

It also remains unknown whether the widespread nocturnality of living birds has been retained from their common ancestors or evolved independently in diverse bird lineages. One recent study has shown that the common ancestor of living birds may have been both diurnal and nocturnal [8], and if this is true, it would suggest that the widespread nocturnality of living birds stems, in part, from their common ancestors. Alternatively, widespread nocturnality may have evolved independently in diverse bird lineages. To distinguish between these two possibilities, it is necessary to reconstruct the evolutionary history of the diel activity patterns within the class Aves.

**Fig. 1:**
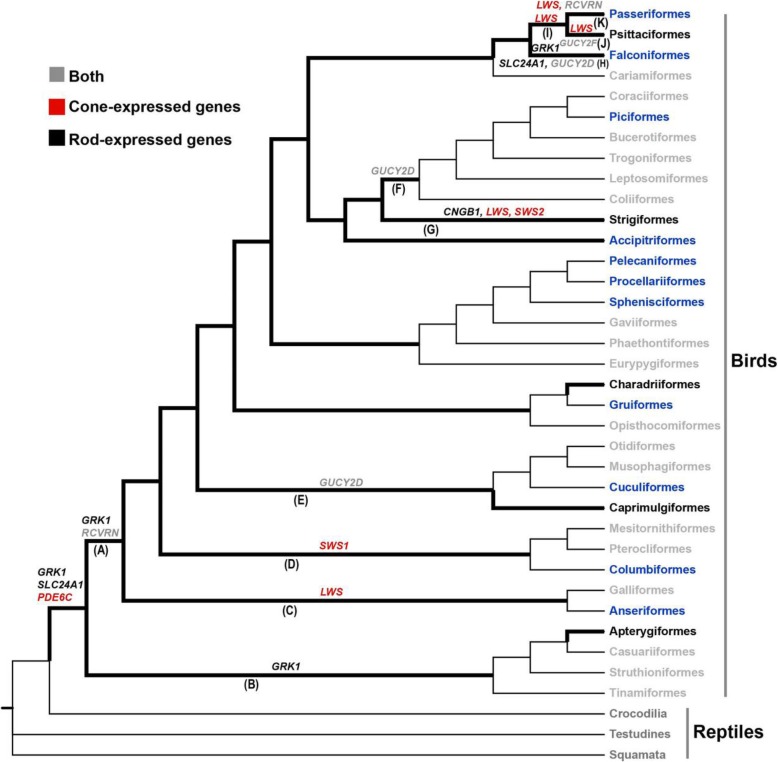
Positively selected genes identified among lineages of birds. The phylogenetic relationships of bird taxa follow previous studies [14–25] and the Tree of Life Web Project (http://tolweb.org/Passeriformes). The diel activity of taxonomic bird orders follows one previous study [1]. The diurnal bird orders that contain taxa with occasional nocturnal activity are shown in blue, bird orders that harbor regular nocturnal taxa are shown in black, and diurnal bird orders with no known nocturnal activity are shown in gray. Please see Additional file 1 for species used for each of the bird orders. Bold lines indicate branches subjected to positive selection analyses in this study. The letters (A-K) in parentheses indicate the branches on which PSGs were detected in this study. The PSGs found are shown along the branches. The three PSGs found along the ancestral bird branch in our recently published study [8] are also shown. Please see the text for details about the positively selected genes identified and their corresponding specific branches

**Fig. 2:**
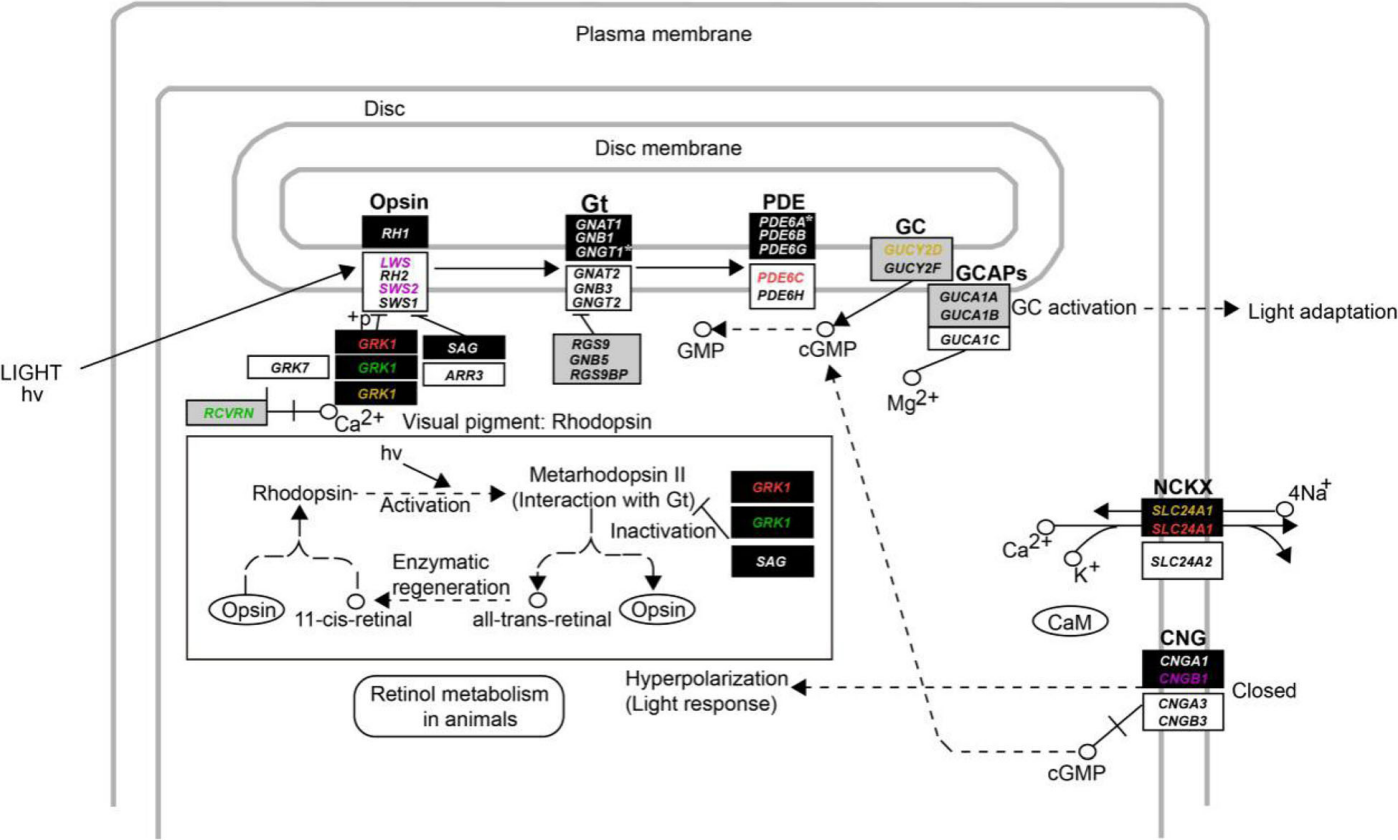
Positively selected genes mapped onto the phototransduction pathway. Only the positively selected genes found in the common ancestor of extant birds (red), ancestral Carinatae (green), falcons (orange) and owls (violet) are shown. For convenience, both the genes involved in the rod phototransduction pathway (according to KEGG pathway map 04744) and the cone phototransduction pathway are shown. The genes that are involved in the phototransduction pathway of rods, cones and both are shown as dark rectangles, white rectangles and gray rectangles, respectively [12, 13]. *Represents two lost rod-expressed genes, *GNGT1* and *PDE6A*, in both reptiles and birds based on previous studies [7, 9]

A recently developed molecular phyloecological (MPE) approach has been demonstrated to reliably reconstruct the diel activity patterns of ancestral taxa [9]. The MPE approach employs phylogenetic analyses of rod and cone phototransduction genes [12, 13], which are specialized for dim-light vision and bright-light vision, respectively, and has revealed that the phylogenetic branches of nocturnal species and diurnal species are mainly characterized by positive selection on rod-expressed genes and cone-expressed genes, respectively [7, 9–11]. Following the MPE approach, we identified evidence for positive selection on two rod-expressed genes, *GRK1* and *SLC24A1*, and one cone-expressed gene, *PDE6C*, in the common ancestor branch of living birds (Fig. 1, Fig. 2), suggesting that ancestral birds were active during the day and night [8]. In this study, we extended our analyses of the same dataset to examine possible diel activity evolution within birds. Our results provide insights into the genetic bases and evolutionary origins of widespread nocturnality in birds.

## Results and discussion

Using the MPE approach to infer the ancestral states of diel activity patterns [8–11], we analyzed the adaptive evolution of 33 phototransduction genes (Additional file 1) among diverse lineages of birds in the sauropsid phylogeny (Fig. 1) using the branch model, the branch-site model and clade model C implemented in PAML software [26]. Positively selected genes (PSGs) were identified using the branch-site model and clade model C, and inferences of positive selection remained robust in terms of the initial variation in kappa and ω values.

Many internal branches of the bird clade exhibited no signals of positive selection, and only partial branches were found to be under positive selection (Fig. 1, Table 1). In Ratitae, we found one rod-expressed gene, *GRK1*, to be under positive selection along the common ancestor branch of the ostrich (*Struthio camelus*) and the emu (*Dromaius novaehollandiae*). *GRK1* is a photoresponse recovery gene involved in the inactivation of activated rhodopsin. As photoresponse recovery is associated with motion detection [27], the positive selection on the photoresponse recovery gene suggests an increased capacity for motion detection in dim-light conditions. For Carinatae, we initially examined the positive selection along the ancestral branch (branch A in Fig. 1) and found two PSGs (*GRK1* and *RCVRN*) (Fig. 2). Both genes are involved in photoresponse recovery, and evidence of positive selection on these genes, which was also supported by a branch-site unrestricted statistical test for episodic diversification (BUSTED), which provides a gene-wide robust test for evidence of selection (Additional file 2), suggests that ancestral Carinatae may have evolved a particularly enhanced capacity for motion detection in at least dim-light conditions. Moreover, the red-sensitive cone opsin gene *LWS* and the ultraviolet/violet-sensitive cone opsin gene *SWS1* were also found to be under positive selection along the *Gallus gallus* and *Columba livia* branches, respectively, suggesting an enhanced ability for bright-light vision in these lineages. One positively selected photoresponse recovery gene, *GUCY2D*, was identified along the branches leading to *Cuculus canorus* and the common ancestor branches of *Upupa epops* and *Picus canus*, respectively. *GUCY2D* encodes guanylyl cyclases, which are involved in the resynthesis of cGMP, promoting photoresponse recovery. We also detected signals of positive selection on the red-sensitive cone opsin gene *LWS* and two photoresponse recovery genes, *RCVRN* and *GUCY2F*, in two closely related groups, *Passeriformes* and *Psittaciformes*, suggesting their increased capacities for motion detection in bright-light conditions.

**Table 1:** Positively selected genes detected based on the PAML branch-site model. For convenience, only the ω values of foreground branches are shown. Only the positively selected sites with a high posterior probability support (> 0.900) are shown

Branches/Genes	Parameter estimates	2∆L	df	*P*-value	Corrected *P*-value	Positively selected sites
(A)						
*GRK1*	*p*_*0*_ = 0.929 *p*_*1*_ = 0.051 *p*_*2a*_ = 0.017 *p*_*2b*_ = 0.000	22.82	1	1.778E-06	2.489E-05	182 S, 269 E
	*ω*_*0*_ = 0.038 *ω*_*1*_ = 1.000 *ω*_*2a*_ = 999.000 *ω*_*2b*_ = 999.000					
*RCVRN*	*p*_*0*_ = 0.915 *p*_*1*_ = 0.022 *p*_*2a*_ = 0.060 *p*_*2b*_ = 0.001	4.46	1	0.034	0.612	5 K, 19 T, 37 R
	*ω*_*0*_ = 0.019 *ω*_*1*_ = 1.000 *ω*_*2a*_ = 28.660 *ω*_*2b*_ = 28.660					
(B)						
*GRK1*	*p*_*0*_ = 0.902 *p*_*1*_ = 0.050 *p*_*2a*_ = 0.044 *p*_*2b*_ = 0.002	5.71	1	0.016	0.224	9 K, 25 K, 252 N, 316 I
	*ω*_*0*_ = 0.038 *ω*_*1*_ = 1.000 *ω*_*2a*_ = 11.732 *ω*_*2b*_ = 11.732					
(C)						
*LWS*	*p*_*0*_ = 0.898 *p*_*1*_ = 0.091 *p*_*2a*_ = 0.008 *p*_*2b*_ = 0.000	7.09	1	0.007	0.112	70 S, 254 S
	*ω*_*0*_ = 0.036 *ω*_*1*_ = 1.000 *ω*_*2a*_ = 40.133 *ω*_*2b*_ = 40.133					
(D)						
*SWS1*	*p*_*0*_ = 0.948 *p*_*1*_ = 0.010 *p*_*2a*_ = 0.040 *p*_*2b*_ = 0.000	4.55	1	0.032	0.512	78 T
	*ω*_*0*_ = 0.023 *ω*_*1*_ = 1.000 *ω*_*2a*_ = 10.955 *ω*_*2b*_ = 10.955					
(E)						
*GUCY2D*	*p*_*0*_ = 0.906 *p*_*1*_ = 0.089 *p*_*2a*_ = 0.003 *p*_*2b*_ = 0.000	6.03	1	0.014	0.126	459 F, 565 K
	*ω*_*0*_ = 0.046 *ω*_*1*_ = 1.000 *ω*_*2a*_ = 302.150 *ω*_*2b*_ = 302.150					
(F)						
*GUCY2D*	*p*_*0*_ = 0.905 *p*_*1*_ = 0.089 *p*_*2a*_ = 0.004 *p*_*2b*_ = 0.000	5.68	1	0.017	0.153	69 K, 161 D
	*ω*_*0*_ = 0.046 *ω*_*1*_ = 1.000 *ω*_*2a*_ = 50.303 *ω*_*2b*_ = 50.303					
(G)						
*CNGB1*	*p*_*0*_ = 0.896 *p*_*1*_ = 0.087 *p*_*2a*_ = 0.014 *p*_*2b*_ = 0.001	3.83	1	0.050	1.150	346 N, 475 G
	*ω*_*0*_ = 0.047 *ω*_*1*_ = 1.000 *ω*_*2a*_ = 6.517 *ω*_*2b*_ = 6.517					
*LWS*	*p*_*0*_ = 0.877 *p*_*1*_ = 0.090 *p*_*2a*_ = 0.028 *p*_*2b*_ = 0.002	16.28	1	5.439E-05	8.702E-04	126 K, 130 R, 237 H
	*ω*_*0*_ = 0.036 *ω*_*1*_ = 1.000 *ω*_*2a*_ = 67.591 *ω*_*2b*_ = 67.591					
*SWS2*	*p*_*0*_ = 0.824 *p*_*1*_ = 0.125 *p*_*2a*_ = 0.042 *p*_*2b*_ = 0.006	10.94	1	0.000	0.013	18 A, 42 K, 43 A
	*ω*_*0*_ = 0.038 *ω*_*1*_ = 1.000 *ω*_*2a*_ = 237.344 *ω*_*2b*_ = 237.344					
(H)						
*GRK1*	*p*_*0*_ = 0.878 *p*_*1*_ = 0.047 *p*_*2a*_ = 0.070 *p*_*2b*_ = 0.003	13.14	1	0.000	0.004	2 L, 9 K, 14 E, 16 C, 25 K, 29 N, 33 N, 44 E
	*ω*_*0*_ = 0.037 *ω*_*1*_ = 1.000 *ω*_*2a*_ = 15.634 *ω*_*2b*_ = 15.634					197 E, 250 R, 289 A, 292 T, 303 K, 306 Y
*GUCY2D*	*p*_*0*_ = 0.903 *p*_*1*_ = 0.088 *p*_*2a*_ = 0.006 *p*_*2b*_ = 0.000	8.35	1	0.003	0.027	175 G
	*ω*_*0*_ = 0.046 *ω*_*1*_ = 1.000 *ω*_*2a*_ = 999.000 *ω*_*2b*_ = 999.000					
*SLC24A1*	*p*_*0*_ = 0.736 *p*_*1*_ = 0.257 *p*_*2a*_ = 0.003 *p*_*2b*_ = 0.001	4.93	1	0.026	0.520	233 I
	*ω*_*0*_ = 0.048 *ω*_*1*_ = 1.000 *ω*_*2a*_ = 42.679 *ω*_*2b*_ = 42.679					
(I)						
*LWS*	*p*_*0*_ = 0.902 *p*_*1*_ = 0.092 *p*_*2a*_ = 0.004 *p*_*2b*_ = 0.000	5.00	1	0.025	0.400	97 S
	*ω*_*0*_ = 0.037 *ω*_*1*_ = 1.000 *ω*_*2a*_ = 58.101 *ω*_*2b*_ = 58.101					
(J)						
*GUCY2F*	*p*_*0*_ = 0.770 *p*_*1*_ = 0.228 *p*_*2a*_ = 0.001 *p*_*2b*_ = 0.000	8.15	1	0.004	0.100	306 E
	*ω*_*0*_ = 0.063 *ω*_*1*_ = 1.000 *ω*_*2a*_ = 244.082 *ω*_*2b*_ = 244.082					
*LWS*	*p*_*0*_ = 0.908 *p*_*1*_ = 0.084 *p*_*2a*_ = 0.006 *p*_*2b*_ = 0.000	4.01	1	0.045	0.720	43 K
	*ω*_*0*_ = 0.038 *ω*_*1*_ = 1.000 *ω*_*2a*_ = 59.283 *ω*_*2b*_ = 59.283					
(K)						
*LWS*	*p*_*0*_ = 0.904 *p*_*1*_ = 0.082 *p*_*2a*_ = 0.011 *p*_*2b*_ = 0.001	6.41	1	0.011	0.176	42 A, 88 L
	*ω*_*0*_ = 0.038 *ω*_*1*_ = 1.000 *ω*_*2a*_ = 213.404 *ω*_*2b*_ = 213.404					
*RCVRN*	*p*_*0*_ = 0.960 *p*_*1*_ = 0.023 *p*_*2a*_ = 0.015 *p*_*2b*_ = 0.000	7.22	1	0.007	0.126	
	*ω*_*0*_ = 0.019 *ω*_*1*_ = 1.000 *ω*_*2a*_ = 999.000 *ω*_*2b*_ = 999.000					

Corrected *P*-value, Bonferroni multiple testing correction, *P* values are corrected by multiplying them by the number of branches tested of each gene. Significance level is *P* < 0.05

Among the groups examined in Carinatae, owls and falcons showed relatively strong positive selection relative to the other groups in the clade. For falcons (branch H in Fig. 1), we found three positively selected photoresponse recovery genes (*GRK1*, *SLC24A1* and *GUCY2D*), two of which (*GRK1* and *SLC24A1*) were rod-expressed genes (Table 1, Fig. 2). In particular, *SLC24A1* encodes the Na^+^/Ca^2+^-K^+^ ion exchanger, extruding free calcium in the outer segment of rods for the restoration of cGMP concentration. The finding of positive selection on these genes suggests that falcons have evolved an enhanced capability to detect motion in dim-light conditions, consistent with the findings of previous studies [7, 28–31]. For owls (branch G in Fig. 1), which are most active at night and during crepuscular periods, we found a marginally significant signal of positive selection on one rod-expressed gene, *CNGB1* (LRT *P*-value = 0.050). *CNGB1* encodes the β subunit of CNG channels and is involved in phototransduction activation. Moreover, two cone opsin genes, the red-sensitive opsin gene *LWS* and the blue-sensitive opsin gene *SWS2*, showed strong signals of positive selection (LRT *P-*value < 0.001) (Table 1, Fig. 2). Positive selection on these two cone opsin genes is associated with spectral tuning to maximize light abortion under crepuscular conditions [7]. When the evidence for positive selection on the PSGs found by PAML in falcons and owls was examined using BUSTED, positive selection on the gene *GRK1* in falcons and the two cone opsin genes *LWS* and *SWS2* in owls was retained (Additional file 2). In addition to the nocturnal owls, we also looked for evidence of positive selection in lineages that contain true nocturnal taxa, including Apterygiformes, Caprimulgiformes and Charadriiformes [1], for which only partial gene sequences were available, but found no evidence of positive selection in these groups. Future studies using retinal transcriptome sequencing would allow us to obtain full-length phototransduction gene sequences and perform detailed analyses of the genes underlying night-vision adaptation in these nocturnal lineages.

In addition to the branch model and branch-site model, we also used clade model C to look for evidence of positive selection on phototransduction genes in birds (Table 2). When the entire clade of birds was analyzed as a foreground clade, we detected relatively strong positive selection signals for both rod-expressed genes (*CNGA1*, *PDE6B*, *SAG* and *SLC24A1*) and cone-expressed genes (*CNGB3*, *PDE6C* and *SLC24A2*), suggesting that both the dim-light vision and bright-light vision of birds were subject to divergent selection compared to those of the reptiles included in the study. This finding is likely a result of the differential adaptation of different bird lineages to their specific light environments [1].

**Table 2:** Positively selected genes of the entire clade of birds based on the clade model C

Genes	Parameter estimates	2∆L	df	*P-value*
*CNGA1*	*p*_*0*_ = 0.903 *p*_*1*_ = 0.086 *p*_*2*_ = 0.010	17.88	1	2.350E-05
	*ω*_*0*_ = 0.041 *ω*_*1*_ = 1.000 *ω*_*2*_ = 2.231			
*CNGB3*	*p*_*0*_ = 0.808 *p*_*1*_ = 0.187 *p*_*2*_ = 0.004	9.80	1	0.001
	*ω*_*0*_ = 0.052 *ω*_*1*_ = 1.000 *ω*_*2*_ = 5.094			
*PDE6B*	*p*_*0*_ = 0.933 *p*_*1*_ = 0.063 *p*_*2*_ = 0.002	4.56	1	0.032
	*ω*_*0*_ = 0.036 *ω*_*1*_ = 1.000 *ω*_*2*_ = 2.620			
*PDE6C*	*p*_*0*_ = 0.851 *p*_*1*_ = 0.136 *p*_*2*_ = 0.011	38.37	1	5.845E-10
	*ω*_*0*_ = 0.048 *ω*_*1*_ = 1.000 *ω*_*2*_ = 3.145			
*SAG*	*p*_*0*_ = 0.806 *p*_*1*_ = 0.177 *p*_*2*_ = 0.015	24.06	1	9.336E-07
	*ω*_*0*_ = 0.077 *ω*_*1*_ = 1.000 *ω*_*2*_ = 2.621			
*SLC24A1*	*p*_*0*_ = 0.737 *p*_*1*_ = 0.249 *p*_*2*_ = 0.013	19.75	1	8.816E-06
	*ω*_*0*_ = 0.050 *ω*_*1*_ = 1.000 *ω*_*2*_ = 4.065			
*SLC24A2*	*p*_*0*_ = 0.782 *p*_*1*_ = 0.193 *p*_*2*_ = 0.024	13.68	1	0.000
	*ω*_*0*_ = 0.051 *ω*_*1*_ = 1.000 *ω*_*2*_ = 2.917			

2∆L: twice the difference of likelihood values between two nested models; df: degrees of freedom; *p*: proportion of sites in different site classes (*p*_*0*_, *p*_*1*_ and *p*_*2*_)

## Conclusions

Nocturnal activity in living birds, which widely occurs in nocturnal and many diurnal avian lineages, has long been documented. In this study, we reconstructed the evolutionary history of the diel activity patterns within birds using a molecular approach. Our results show that the common ancestor of living birds and ancestral Carinatae was considerably active in dim-light conditions, suggesting a deep evolutionary origin of nocturnality in birds. Further analyses indicated that the diel activity pattern of birds may have remained relatively unchanged with the subsequent diversification of most bird lineages, suggesting that the widespread nocturnality of living birds was more likely retained from their common ancestors than independently derived. The phylogenetic analyses of phototransduction genes show that functional enhancement of the two rod-expressed genes *GRK1* and *SLC24A1* underlies the widespread nocturnality of living birds. Moreover, photoresponse recovery genes were found to be frequently subjected to positive selection in diverse bird lineages, suggesting that birds have widely evolved an enhanced ability to detect motion.

## Methods

### Taxa and sequences used

The retinal transcriptome data of 17 bird species published in our previous studies [7, 8], along with data from all of the other reptiles and birds with genomes available in GenBank, were used in this study (Additional file 1). In total, 95 species were used, including 80 bird species from 34 orders, representing the majority of living bird orders (34/39) [32]. For the 95 species used, the coding sequences of 33 phototransduction genes were obtained. We used the online software webPRANK (http://www.ebi.ac.uk/goldman-srv/webprank/) for sequence alignment, and individual sequences with low identities, long indels, multiple ambiguous bases (Ns), and/or too short a length were removed or replaced with other relevant transcript variants. After this pruning, high-quality alignments were constructed, and their translated protein sequences were blasted against the nonredundant protein sequence database to confirm that the sequences were trimmed and/or inferred correctly.

### Positive selection analyses

We analyzed our focal genes for evidence of positive selection using the branch model, the branch-site model and clade model C implemented in the Codeml program of PAML [26]. Thus, a codon-based maximum likelihood method was used to estimate the ratio of nonsynonymous to synonymous substitutions per site (dN/dS or ω), and likelihood ratio tests (LRTs) were employed to determine whether the results were statistically significant. A statistically significant value of ω > 1 suggested positive selection. *P* values were subjected to Bonferroni correction. Upon analysis, we constructed an unrooted species tree following published studies [14–25] and the Tree of Life Web Project (http://tolweb.org/Passeriformes). The phylogenetic relationships among bird orders followed one genome-level study [18].

#### Branch model

We used a two-rate branch model to detect possible positive selection signals along the branches of interest. For the analyses, each of our focal branches was designated as the foreground branch, and the others were treated as background branches. In the two-rate branch model, foreground branches and background branches were assumed to have different ω values, and the goodness of fit of the two-rate branch model was analyzed using an LRT. Using an LRT, the two-rate branch model was compared with the one-rate branch model that assumed a single ω value across the tree, and if a statistically significant value of ω > 1 was detected in a foreground branch, the two-rate branch model was further compared with a two-rate branch model in which ω was constrained on all branches to ω = 1 to further assess whether the ω > 1 of the foreground branch was statistically supported.

#### Branch-site model

We used a branch-site model (Test 2) to look for evidence of positively selected sites on our focal branches. For the analyses, four classes of sites were assumed. Site class 0 (0 < ω0 < 1) and site class 1 (ω1 = 1) were assumed to represent evolutionarily conserved and neutral codons, respectively, for both background branches and foreground branches. Site classes 2a and 2b were assumed to represent evolutionarily conserved (0 < ω0 < 1) and neutral (ω1 = 1) codons, respectively, for background branches but were allowed to be under positive selection (ω2 > 1) on the foreground branches. In the branch-site model, a modified model A was compared with its corresponding null model with ω = 1 constrained to determine the statistical significance. The empirical Bayes method was used to detect positively selected sites.

#### Clade model C

We used clade model C to examine evidence for possible divergent selection on phototransduction genes in birds. To this end, the entire bird clade was designated as the foreground clade, while the others were treated as the background clades. Model C assumed three site classes, where site class 0 (0 < ω0 < 1), site class 1 (ω1 = 1) and site class 2 (ω2, 3) represented evolutionarily conserved, evolutionarily neutral and potentially positively selected codons, respectively, in both the background and foreground clades. Clade model C compares model C with its corresponding null model M2a_rel to determine LRT statistical significance.

### Robustness test of positively selected genes

To test the robustness of our results, we examined the dependency of our positively selected genes on the initial values of kappa and omega. Thus, two different initial values of kappa (kappa = 0.5, 3.0) and omega (ω = 0.5, 2.0) were used for the positive selection analyses, and four independent runs were conducted for each of the positively selected genes. In addition, we used BUSTED, implemented in HyPhy software (version 2.2.4) [33, 34], to confirm the positively selected genes identified by the branch-site model of PAML. BUSTED differs from PAML in that it allows for the occurrence of positive selection on both foreground and background branches (unconstrained model), while PAML allows positive selection only on foreground branches. The fit of the unconstrained model was compared with that of the null model that disallowed ω > 1 among the foreground branches. Statistical significance was determined by an LRT.

## Supplementary information

**Additional file 1.** Species and sequences used in this study.

**Additional file 2.** Positive selection analyses using BUSTED.

## Data Availability

All data needed to evaluate the conclusions in the paper are present in the paper and/or the additional files.

## Abbreviations

LRT: Likelihood ratio tests
MPE: Molecular phyloecological
