# Supplementary material for: Widespread nocturnality of living birds stemming from their common ancestor
Source: BMC Evol Biol. 2019 Oct 16;19:189. doi: 10.1186/s12862-019-1508-y (PMC6794809; doi:10.1186/s12862-019-1508-y)
Supplement: Supplementary file 2 — Additional file 2. Positive selection analyses using BUSTED. [file 12862_2019_1508_MOESM2_ESM.docx]

**Additional file 2. Positive selection analyses using BUSTED.** Only the positively selected genes of ancestral branches of Carinatae, owls and falcons identified by the branch-site model of PAML were examined. For analyses, the ancestral branches of three groups were respectively treated as foreground branches and others were used as background branches.

| **Branch/Gene** | **Model** | **log (L)** | **ω3 (Proportion)** | ***P-value*** |
| --- | --- | --- | --- | --- |
| **(A)** |  |  |  |  |
| *GRK1* |  |  |  |  |
|  | Unconstrained | -8186.82 | 1466.46 (1.89%) | **0.000^***^** |
|  | Constrained | -8195.79 |  |  |
| *RCVRN* |  |  |  |  |
|  | Unconstrained | -3416.48 | 83.74 (6.89%) | 0.055 |
|  | Constrained | -3419.37 |  |  |
| **(G)** |  |  |  |  |
| *CNGB1* |  |  |  |  |
|  | Unconstrained | -20099.17 | 2.67 (9.37%) | 0.533 |
|  | Constrained | -20099.80 |  |  |
| *LWS* |  |  |  |  |
|  | Unconstrained | -7408.56 | 9.82 (8.92%) | **0.000^***^** |
|  | Constrained | -7416.92 |  |  |
| *SWS2* |  |  |  |  |
|  | Unconstrained | -2847.87 | 177.93 (7.31%) | **0.006^**^** |
|  | Constrained | -2852.91 |  |  |
| **(H)** |  |  |  |  |
| *GRK1* |  |  |  |  |
|  | Unconstrained | -8187.67 | 9.67 (7.20%) | **0.000^***^** |
|  | Constrained | -8195.85 |  |  |
| *GUCY2D* |  |  |  |  |
|  | Unconstrained | -17161.99 | 2.52 (7.51%) | 0.288 |
|  | Constrained | -17163.24 |  |  |
| *SLC24A1* |  |  |  |  |
|  | Unconstrained | -17684.16 | 26.55 (0.97%) | 0.093 |
|  | Constrained | -17686.53 |  |  |

***P < 0.01, ***P < 0.001*
